# Factors Associated With Non-participation in a Face-to-Face Second Survey Conducted 5 Years After the Baseline Survey

**DOI:** 10.2188/jea.JE20140116

**Published:** 2015-02-05

**Authors:** Megumi Hara, Chisato Shimanoe, Yasuko Otsuka, Yuichiro Nishida, Hinako Nanri, Mikako Horita, Jun Yasukata, Nobuyuki Miyoshi, Yosuke Yamada, Yasuki Higaki, Keitaro Tanaka

**Affiliations:** 1 Department of Preventive Medicine, Faculty of Medicine, Saga University, Saga, Japan; 1 佐賀大学医学部社会医学講座予防医学; 2 Department of Public Health, Showa University, Tokyo, Japan; 2 昭和大学医学部衛生学公衆衛生学講座公衆衛生学; 3 Laboratory of Exercise Physiology, Faculty of Sports and Health Science, Fukuoka University, Fukuoka, Japan; 3 福岡大学スポーツ科学部; 4 Department of Childhood Care Education, Seika Women’s Junior College, Fukuoka, Japan; 4 精華女子短期大学幼児保育学科; 5 Department of Nutritional Science, National Institute of Health and Nutrition, Tokyo, Japan; 5 国立健康・栄養研究所基礎栄養研究部エネルギー代謝研究室

**Keywords:** non-participation, second survey, cohort, socioeconomic factors, lifestyle-related factors

## Abstract

**Background:**

Non-participation in second surveys is reported to be associated with certain baseline characteristics; however, such data are unavailable for Japanese populations. Although disease incidence during follow-up might influence participation, few reports have addressed this possibility. This study sought to identify factors associated with non-participation in a second survey of a population-based cohort, and to evaluate the influence of self-reported disease incidence on non-participation.

**Methods:**

After excluding participants who left the area (*n* = 423), died (*n* = 163), and withdrew from the study (*n* = 9) among 12 078 participants in a baseline survey for the Japan Multi-Institutional Collaborative Cohort Study in the Saga region between 2005 and 2007, 11 483 people were invited by mail to participate in a face-to-face second survey between 2010 and 2012. The 5-year clinical health history of non-participants was assessed by mail or telephone. Baseline characteristics and self-reported clinical outcomes of non-participants were compared with those of participants.

**Results:**

Among 11 483 people, 8454 (73.6%) participated in the second survey, and 2608 out of 3029 non-participants answered mail or telephone health surveys. Female sex, youngest and oldest ages, lower education, lower occupational class, current smoking, lower physical activity level, shorter sleep time, obesity, and constipation were associated with non-participation. Light drinking (0.1–22.9 g ethanol/day) was associated with participation. Non-participants reported a significantly higher incidence of cancer and a significantly lower proportion of hypertension compared with participants.

**Conclusions:**

Both baseline characteristics and disease incidence during the follow-up period had significant associations with non-participation in the face-to-face second survey.

## Abstract (ja)

**背景：:**

第二次調査への参加状況はベースライン時点の特性と関連があると報告されているが、日本人についての報告はない。また、追跡期間中の疾患罹患も第二次調査への参加状況に影響すると考えられるが、報告は少ない。本研究では、住民ベースのコーホート研究の第二次調査への非参加に関連するベースライン時点の要因を明らかにするとともに、追跡期間中の疾病罹患の影響について評価した。

**方法：:**

2005年から2007年にかけて実施された日本多施設共同コーホート研究佐賀地区に参加した12,078人の参加者から、追跡期間中の転出（423人）、死亡（163人）、同意撤回（9人）を除いた11,483人を対象に、2010年から2012年にかけて対面式の第二次調査を実施した。非参加者については、郵送法または電話で5年間の疾患罹患を調査した。非参加者のベースライン時点の特性、および自己申告の追跡期間中の疾患罹患状況について参加者と比較した。

**結果：:**

11,483人のうち8,454人（73.6％）が第二次調査に参加し、非参加者3,029人のうち2,608人が郵送法または電話で罹患状況について回答した。ベースライン調査時点の性（女性）、年齢（若い群と最も高齢群）、短い教育歴、低い職業クラス、喫煙、低い身体活動レベル、短い睡眠時間、肥満、便秘が第二次調査への非参加と、適量飲酒（１日エタノール摂取量23ｇ未満）が参加と関連していた。非参加者は参加者に比べて、追跡期間中のがん罹患割合が高く、高血圧の発症割合が低かった。

**結論：:**

ベースライン調査時点の特性と追跡期間中の罹患状況の両方が、対面式の第二次調査への参加状況と有意に関連していた。

## INTRODUCTION

Study participants failing to take part in follow-up (second) surveys has the potential to reduce the power of the study, can bias the findings, and can reduce the generalizability of the results.^1^ High rates of non-participation in follow-up surveys can lead to selection bias when differences in characteristics between non-participants and participants are related to the outcome being studied. Various longitudinal studies have compared baseline characteristics between participants and non-participants in second surveys. The results suggest that non-participants are more likely to be the youngest of the adult category,^2^^–^^6^ the oldest of the elderly category,^7^^,^^8^ to live alone,^3^^–^^5^ to be less educated,^2^^,^^4^^,^^7^^,^^9^^–^^11^ to have an unhealthy lifestyle,^2^^–^^4^^,^^7^^,^^9^^,^^10^^,^^12^ to be obese,^3^^–^^5^^,^^7^ to have a poor health status,^3^^,^^4^^,^^6^^,^^7^^,^^9^ and to suffer from mental health issues.^5^ The association between these factors and participation differs according to the survey method employed; for example, whether mail, telephone, or face-to-face surveys are used. The association between these baseline characteristics and participation is also influenced by the interval of time elapsed since the baseline survey, study populations, and participation rates. The incidence of disease during follow-up might also influence participation. It has been reported that participants who developed cancer during the follow-up period were less likely to respond to second surveys.^4^^,^^10^ However, to the best of our knowledge, Japanese studies of this type have never been reported. The assessment of determinants of non-participation in second surveys is very important, as it can provide management strategies for future studies and can help evaluate the possibility of bias on the results.

The Japan Multi-Institutional Collaborative Cohort (J-MICC) Study is an on-going cohort study that aims to provide basic data for the prevention of lifestyle-related disease, mainly cancer, by examining the relationship between disease and combinations of lifestyle, genetic traits, and blood component.^13^ We started a population-based cohort study in the Saga region as part of the J-MICC Study, and a face-to-face baseline survey was completed with 12 078 participants (from an eligible population of about 62 000 adults aged 40 to 69 years) recruited between 2005 and 2007.^14^ Results from our previous study suggested that being female, being older, and having easy access to survey locations were associated with a higher participation rate in the baseline survey.^14^ The cohort is a selected sample with an increased interest in health, and such participants may be more motivated to participate in a second survey than the general population; however, it is unknown which factors at baseline and follow-up are associated with the participation rate of the second survey conducted 5 years after the baseline survey.

The purposes of the current study were to document the method used for the second survey of the J-MICC Study’s Saga region cohort and to clarify factors associated with non-participation in that study.

## METHODS

### Baseline survey and follow-up

The J-MICC Study’s Saga region cohort was established from 2005 to 2007. The details of the baseline survey have been previously reported.^14^ Briefly, a total of 61 447 residents in Saga City who were 40–69 years of age were invited by mail to participate in the study, and the survey date and time were arranged by telephone. In total, 12 078 people participated in the baseline survey. Participation involved answering a questionnaire that included items on age, gender, education level, occupational status, lifestyle-related factors, past medical history, and medication status. Blood pressure (systolic and diastolic) and anthropometric characteristics (height, weight, body fat percentage, and waist and hip circumferences) were recorded, and a 21-mL blood sample was taken. Participants who agreed to allow measurements of their daily physical activities wore accelerometers (Lifecorder; Suzuken Co., Ltd., Nagoya, Japan) for 10 days and then returned them by mail.

The cohort is to be followed until 2025, and participants are to be removed from the study if they move out of Saga City. Information on date of leaving the city, along with any new address and date of death, if appropriate, will be obtained by reviewing the population registry of Saga City every year. When it is confirmed that a participant has left the city, they are sent a health survey questionnaire to gather information on disease incidence up until their departure. Reminders are sent once or twice to non-responders. Cause of death is confirmed by viewing death certificates at the Saga-chubu Public Health and Welfare Office, with the permission of the Director-General of the Prime Minister’s Office (Ministry of Public Management, Home Affairs, Posts and Telecommunications). During the 5 years between the baseline and the second surveys, 423 people left the study area, 163 deaths were recorded, and 9 people withdrew from the study. The remaining 11 483 people were considered the target population for the 5-year second survey (Figure).

**Figure.**
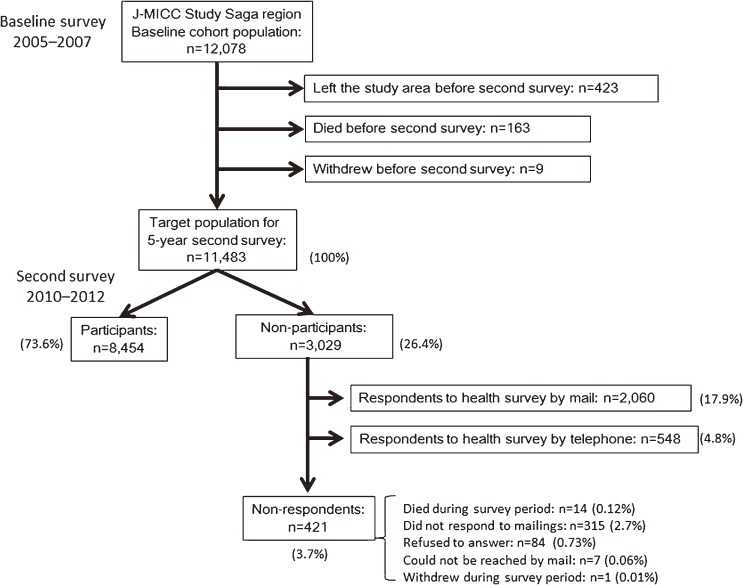
Flow of participants in the J-MICC Study’s Saga region cohort.

### Face-to-face second survey 5 years after baseline survey

Cohort participants were sent a newsletter every March to inform them of the progress of the study, to keep in touch, and to notify them of the face-to-face 5-year second survey beginning in 2010. The second survey was conducted from 1 November 2010 until 25 November 2012 in 19 areas defined by public elementary school attendance boundaries. The survey was administered during the day, as was the baseline survey,^14^ usually on two weekdays and one weekend, in a public hall within residential areas of the participants and other halls available within or outside the areas. One month before the survey was to commence, an invitation letter containing an explanation of the study, a schedule of second surveys, and a request for participation was sent to the target population, and they were asked to reply by mail or fax if they chose to participate. Two weeks following the initial invitation letter a reminder was sent to non-responders. A schedule of the days of the investigation was arranged by telephone with all who chose to participate. A self-administered questionnaire, which included almost the same questions as those contained in the baseline survey, and a health survey, which asked about the incidence of diseases, including hypertension, diabetes, stroke, ischemic heart disease, and cancer during the 5 years since the baseline survey, were sent to participants prior to the study. Participants were asked to bring their completed questionnaires to the study site.

An oral explanation of the purpose, content, and conditions of cooperation of the study was provided to participants, and participants were asked to fill out informed consent forms. Following inspection of completed questionnaires by research nurses, participants’ blood pressure and anthropometric characteristics were measured, and a 21-mL blood sample was taken. Participants were also asked to provide a 20-mL urine sample taken immediately upon waking.

### Health survey to non-participants

A health survey was conducted to gather information on disease incidence among those who did not participate in the face-to-face second survey. The health survey questionnaire was sent just after the second survey to non-participants in every area. A reminder was sent 2 weeks later to non-responders. When the second survey was completed in all areas, the health survey questionnaire was again sent to continued non-responders. If they still failed to respond, information on their health status was gathered by telephone.

### Analyses

To compare baseline characteristics between participants and non-participants, we chose determinants of socioeconomic variables. These included gender (male or female), age (40–44, 45–49, 50–54, 55–59, 60–64, or 65–69 years), educational level (≤12 or >12 years), occupational class (high: armed forces, managers, technicians, and associate professionals or professionals; medium: clerical support workers or service and sales workers; low: skilled agricultural, forestry and fishery workers, craft and related trades workers, plant and machine operators, and assemblers, or elementary occupations; or none: no job, housekeeper, or not categorized). Lifestyle-related variables included drinking habit (never, former, or current drinker consuming 0.1–22.9, 23.0–45.9, or ≥46 g ethanol/day), smoking habit (never, former, or current), quartiles of physical activity level (calculated as total daily energy expenditure divided by basal energy expenditure: <1.402, 1.402–1.449, 1.450–1.505, or ≥1.506), and hours of sleep (<6, 6 to <7, 7 to <8, or ≥8 hours). Health-related variables included body mass index (BMI; <18, ≥18 to <25, or ≥25 kg/m^2^), perceived mental stress (high, medium, or low),^15^ medical history (cancer, stroke, ischemic heart disease, hypertension, diabetes, and dyslipidemia), and medication status (use of anti-inflammatory drugs or drugs for treating hypertension, diabetes, dyslipidemia, constipation, or sleep disorder). Among the 11 483 study participants, data were missing for occupational class (*n* = 76), alcohol consumption (*n* = 10), smoking status (*n* = 1), physical activity level (*n* = 168), hours of sleep (*n* = 5), BMI (*n* = 13), and perceived stress (*n* = 7). Logistic regression models were used to adjust for potential confounders, and the odds ratios (ORs) and 95% confidence intervals (CIs) were calculated as measures of the strength of association. We also compared the self-reported incidence of disease during the 5 years’ follow-up between participants and non-participants who answered the health survey by mail or telephone. When the 95% CI did not include 1, the corresponding ORs were considered statistically significant at a significance level of 0.05. Analyses were performed using the SAS statistical software package (Ver. 9.3 for Windows; SAS Institute, Cary, NC, USA).

## RESULTS

In total, 11 483 people were invited to participate in the 5-year second survey for the J-MICC Study’s Saga region cohort. The flow of participants through the baseline and second surveys is shown in Figure. Of that population, 8454 people participated in the face-to-face second survey (participation rate: 73.6%). Of the 3029 non-participants, 2060 responded to the health survey by mail, and 548 responded by telephone. No data on disease incidence were obtained from 421 individuals who died during the survey period (*n* = 14), did not respond to mailings (*n* = 315), refused to answer the health survey (*n* = 84), could not be reached by mail (*n* = 7), or withdrew from study participation (*n* = 1). Data from the person who withdrew from the study were excluded from further analyses. Ultimately, baseline characteristics of 3028 non-participants and 8454 participants were compared.

Table 1 shows baseline characteristics of non-participants and participants in the second survey. Non-participants were more likely to be female, younger (among those <60 years old at baseline) or older (among those ≥60 years old at baseline), less educated, of a lower occupational class, current smokers, less physically active, obese, on medication for constipation, and to sleep for shorter periods. Light-to-moderate drinking habit (0.1–22.9 g ethanol/day) was associated with participation. Stratified analysis by gender revealed some differences in the above associations between males and females (eTable 1). For males, compared with 7 hours, both shorter sleep period and longer sleep period were significantly associated with non-participation. For females, non-participants were more likely to be older than 65 years at the baseline survey, of a lower occupational class, obese, medicated for hypertension, and to have a medical history of diabetes. A heavy drinking habit (≥46 g ethanol/day) was associated with non-response in females.

**Table 1.** Baseline characteristics of 11 482 participants in the J-MICC Study’s Saga region cohort by participation status in the follow-up survey, and odds ratios and 95% confidence intervals of non-participation according to these characteristics

Baseline characteristics		Non-participants (*n* = 3028)	Participants (*n* = 8454)	Adjusted OR^a^	(95% CI)
Female (vs male)		1769	4958	1.21	(1.07–1.38)

Age category	40–44	414	968	1.63	(1.38–1.92)
	45–49	439	1017	1.63	(1.38–1.92)
	50–54	491	1345	1.33	(1.14–1.55)
	55–59	571	1774	1.15	(1.00–1.32)
	60–64	524	1783	1.00	(ref)
	65–69	589	1567	1.23	(1.06–1.41)

Education, years (≤12 vs >12)		1826	4605	1.24	(1.13–1.37)

Occupational class	High	741	2199	1.00	(ref)
	Medium	848	2412	1.02	(0.90–1.17)
	Low	459	1042	1.26	(1.09–1.46)
	None	957	2748	1.03	(0.90–1.17)

Drinking status	Never	1267	3329	1.00	(ref)
	Former	102	215	1.01	(0.77–1.31)
	0.1–22.9 g ethanol/day	988	3185	0.84	(0.76–0.93)
	23.0–45.9 g ethanolday	344	933	0.94	(0.80–1.11)
	≥46 g ethanol/day	324	785	0.95	(0.80–1.13)

Smoking status	Never	1712	5278	1.00	(ref)
	Former	544	1667	1.11	(0.97–1.28)
	Current	772	1508	1.59	(1.39–1.81)

Quartiles of PAL	Q1 (<1.402)	854	1913	1.49	(1.31–1.69)
	Q2 (1.402–1.449)	749	2137	1.21	(1.07–1.37)
	Q3 (1.450–1.505)	675	2097	1.11	(0.98–1.26)
	Q4 (≥1.506)	670	2219	1.00	(ref)

Sleeping category (hours)	<6	416	1015	1.18	(1.02–1.36)
	≥6 to <7	1031	2973	1.03	(0.93–1.15)
	≥7 to <8	1035	3083	1.00	(ref)
	≥8	543	1381	1.12	(0.98–1.27)

BMI category (kg/m^2^)	<18.5	185	515	0.97	(0.80–1.16)
	≥18.5 to <25	2009	6110	1.00	(ref)
	≥25	829	1821	1.36	(1.23–1.51)

Percieved stress	High	936	2404	1.05	(0.93–1.19)
	Medium	1419	4072	0.99	(0.88–1.10)
	Low	671	1973	1.00	(ref)

Medical history (vs no history)	Hypertension	637	1583	1.05	(0.86–1.28)
	Diabetes	229	508	1.25	(0.95–1.67)
	Dyslipidemia	570	1648	0.98	(0.85–1.12)
	Ischemic heart disease	93	263	0.87	(0.67–1.13)
	Stroke	58	124	1.17	(0.83–1.64)
	Cancer	164	448	1.05	(0.86–1.27)

On medication (vs no medication)	Hypertension	526	1285	1.11	(0.90–1.38)
	Diabetes	152	333	1.00	(0.71–1.40)
	Dyslipidemia	295	822	0.97	(0.81–1.17)
	Anti-inflammatory drugs	150	335	1.15	(0.93–1.42)
	Constipation	212	423	1.31	(1.09–1.57)
	Sleeping disorder	166	352	1.16	(0.95–1.42)

BMI, body mass index; CI, confidence interval; OR, odds ratio; PAL, physical activity level, which was calculated as total daily energy expenditure divided by basal energy expenditure and classified into quartiles.

^a^Adjusted for all items listed in the table.

Table 2 shows ORs for non-participation in the face-to-face second survey according to the self-reported incidences of disease during the 5 years of follow-up. Rates of self-reported cancer were significantly higher among non-participants than participants in both males and females. In contrast, rates of self-reported hypertension were significantly lower among non-participants than participants, especially in males. The other diseases, such as diabetes, heart disease and stroke, were not associated with participation.

**Table 2.** Self-reported disease incidence during 5-year follow-up by participation status in the face-to-face follow-up survey, and odds ratios and 95% confidence intervals of non-participation according to these diseases

	All (*n* = 11 062)						Male (*n* = 4575)						Female (*n* = 6487)					
		
	Non-participants (*n* = 2608)		Participants (*n* = 8454)		OR^a^	(95% CI)	Non-participants (*n* = 1079)		Participants (*n* = 3496)		OR^a^	(95% CI)	Non-participants (*n* = 1529)		Participants (*n* = 4958)		OR^a^	(95% CI)
					
	*n*	(%)	*n*	(%)			*n*	(%)	*n*	(%)			*n*	(%)	*n*	(%)		
Hypertension	167	(6.4)	704	(8.3)	0.77	(0.64–0.92)	77	(7.1)	371	(10.6)	0.66	(0.51–0.87)	90	(5.9)	333	(6.7)	0.88	(0.69–1.13)
Diabetes	61	(2.3)	225	(2.7)	0.82	(0.61–1.10)	35	(3.2)	132	(3.8)	0.84	(0.37–1.62)	26	(1.7)	93	(1.9)	0.74	(0.47–1.17)
Heart disease	69	(2.7)	249	(3.0)	0.87	(0.66–1.16)	39	(3.6)	153	(4.4)	0.81	(0.56–1.18)	30	(2.0)	96	(1.9)	0.94	(0.61–1.44)
Stroke	41	(1.6)	108	(1.3)	1.15	(0.79–1.68)	25	(2.3)	63	(1.8)	1.25	(0.76–2.06)	16	(1.1)	45	(0.9)	1.08	(0.60–1.95)
Cancer	161	(6.2)	287	(3.4)	1.88	(1.53–2.32)	80	(7.4)	142	(4.1)	2.08	(1.54–2.80)	81	(5.3)	145	(2.9)	1.77	(1.32–2.37)
Any	445	(17.1)	1388	(16.4)	1.03	(0.92–1.17)	220	(20.4)	742	(21.2)	0.96	(0.81–1.15)	225	(14.7)	646	(13.0)	1.10	(0.93–1.31)

CI, confidence interval; OR, odds ratio.

^a^Adjusted for age (5-year increments), education (≤12 years or >12 years), occupational class (high, medium, low, or none), ethanol intake (never, former, or current drinker of 0.1–22.9, 23.0–45.9, or ≥46 g ethanol/day), smoking status (never, former, or current smoker), physical activity level (quartile), hours of sleep (<6, 6–6.9, 7–7.9, or ≥8 hours/day), obesity, percieved stress (high, medium, or low), medical history of hypertension, diabetes, dyslipidemia, ischemic heart disease, stroke, or cancer, and use of medication for hypertension, diabetes, dyslipidemia, inflammation, constipation, or sleep disorder.

Baseline characteristics of health survey participants among non-participants in the face-to-face second survey were compared according to the means by which a participant responded (ie, mail or telephone; Table 3). People who responded by telephone were more likely to be younger (<50 years old at baseline) and obese. They were less likely to be female, to not have an occupation, to have a heavy drinking habit (≥46 g ethanol/day), and to have lower BMI. eTable 2 shows the results of stratified analysis by gender. Associations between means of response and age, education level, occupational class, drinking habit, and obesity were found to be significant in males. Associations between means of response and smoking, lower BMI, and medical history of diabetes were found to be significant in females.

**Table 3.** Baseline characteristics of 2608 people who participated in the health survey but not the face-to-face follow-up survey by response status (mail or telephone), and odds ratios and 95% confidence intervals of telephone responses according to these characteristics

Baseline characteristics		Telephone survey (*n* = 548)	Mail survey (*n* = 2060)	Adjusted OR^a^	(95% CI)
Female (vs male)		260	1269	0.61	(0.45–0.81)

Age category	40–44	110	231	2.28	(1.57–3.32)
	45–49	92	274	1.63	(1.12–2.37)
	50–54	95	338	1.32	(0.93–1.90)
	55–59	105	401	1.23	(0.87–1.72)
	60–64	77	380	1.00	(ref)
	65–69	69	436	0.80	(0.55–1.16)

Education, years (≤12 vs >12)		331	1245	1.21	(0.97–1.50)
					
Occupational class	High	164	467	1.00	(ref)
	Medium	153	578	0.82	(0.62–1.08)
	Low	103	297	1.09	(0.79–1.51)
	None	126	702	0.73	(0.53–0.99)

Drinking status	Never	219	867	1.00	(ref)
	Former	17	67	0.85	(0.47–1.53)
	0.1–22.9 g ethanol/day	169	691	0.79	(0.62–1.01)
	23.0–45.9 g ethanolday	74	224	0.75	(0.53–1.07)
	≥46 g ethanol/day	68	209	0.66	(0.45–0.96)

Smoking status	Never	260	1219	1.00	(ref)
	Former	105	373	0.98	(0.71–1.34)
	Current	183	468	1.29	(0.97–1.70)

Quartiles of PAL	Q1 (<1.402)	143	588	1.10	(0.82–1.47)
	Q2 (1.402–1.449)	136	519	1.11	(0.84–1.48)
	Q3 (1.450–1.505)	127	451	1.19	(0.89–1.60)
	Q4 (≥1.506)	127	463	1.00	(ref)

Sleeping category (hours)	<6	71	272	1.00	(072–1.39)
	≥6 to <7	211	680	1.15	(0.91–1.46)
	≥7 to <8	179	718	1.00	(ref)
	≥8	85	389	0.91	(0.67–1.25)

BMI category (kg/m^2^)	<18.5	16	130	0.52	(0.29–0.90)
	≥18.5 to <25	344	1409	1.00	(ref)
	≥25	187	518	1.43	(1.15–1.79)

Percieved stress	High	179	613	1.11	(0.83–1.50)
	Medium	262	966	1.16	(0.89–1.52)
	Low	107	479	1.00	(ref)

Medical history (vs no history)	Hypertension	104	455	0.68	(0.43–1.07)
	Diabetes	47	138	1.66	(0.89–3.13)
	Dyslipidemia	111	395	1.15	(0.85–1.57)
	Ischemic heart disease	13	62	0.94	(0.50–1.79)
	Stroke	10	40	1.18	(0.56–2.47)
	Cancer	23	114	0.92	(0.57–1.50)

On medication (vs no medication)	Hypertension	89	374	1.36	(0.84–2.24)
	Diabetes	30	95	0.78	(0.36–1.66)
	Dyslipidemia	50	209	0.95	(0.61–1.47)
	Anti-inflammatory drugs	29	94	1.35	(0.85–2.13)
	Constipation	29	149	0.90	(0.58–1.40)
	Sleeping disorder	22	115	0.89	(0.53–1.47)

BMI, body mass index; CI, confidence interval; OR, odds ratio; PAL, physical activity level, which was calculated as total daily energy expenditure divided by basal energy expenditure and classified into quartiles.

^a^Adjusted for all items listed in the table.

Table 4 shows ORs for responses by telephone (vs mail) according to the self-reported incidence of diseases during the 5 years of follow-up. Self-reported incidence of most diseases did not show a statistically significant relationship to the means of response, although self-reported stroke in females showed a significant association to response by telephone.

**Table 4.** Self-reported disease incidence during 5-year follow-up by means of health survey, and odds ratios and 95% confidence intervals of telephone survey according to these diseases among 2608 people who participated in the health survey but not the face-to-face survey

	All (*n* = 2608)						Male (*n* = 1079)						Female (*n* = 1529)					
		
	Telephone (*n* = 548)		Mail (*n* = 2060)		OR^a^	(95% CI)	Telephone (*n* = 288)		Mail (*n* = 791)		OR^a^	(95% CI)	Telephone (*n* = 260)		Mail (*n* = 1269)		OR^a^	(95% CI)
					
	*n*	(%)	*n*	(%)			*n*	(%)	*n*	(%)			*n*	(%)	*n*	(%)		
Hypertension	28	(5.1)	139	(6.8)	0.77	(0.50–1.19)	17	(5.9)	60	(7.6)	0.70	(0.38–1.29)	11	(4.2)	79	(6.2)	0.76	(0.39–1.49)
Diabetes	16	(2.9)	45	(2.2)	1.11	(0.60–2.08)	10	(3.5)	25	(3.2)	0.92	(0.39–2.14)	6	(2.3)	20	(1.6)	1.54	(0.55–4.33)
Heart disease	15	(2.7)	54	(2.6)	1.04	(0.25–1.10)	10	(3.5)	29	(3.7)	0.93	(0.40–2.14)	5	(1.9)	25	(2.0)	0.99	(0.34–2.91)
Stroke	11	(2.0)	30	(1.5)	1.53	(0.71–3.31)	3	(1.0)	22	(2.8)	0.28	(0.06–1.41)	8	(3.1)	8	(0.6)	7.25	(2.30–22.86)
Cancer	29	(5.3)	132	(6.4)	0.91	(0.59–1.41)	12	(4.2)	68	(8.6)	0.73	(0.37–1.42)	17	(6.5)	64	(5.0)	1.29	(0.70–2.36)
Any	87	(15.9)	358	(17.4)	0.93	(0.71–1.22)	46	(16.0)	174	(22.0)	0.78	(0.53–1.15)	41	(15.8)	184	(14.5)	1.14	(0.77–1.69)

CI, confidence interval; OR, odds ratio.

^a^Adjusted for age (5-year increments), education (≤12 years or >12 years), occupational class (high, medium, low, or none), ethanol intake (never, former, or current drinker of 0.1–22.9, 23.0–45.9, or ≥46 g ethanol/day), smoking status (never, former, or current smoker), physical activity level (quartile), hours of sleep (<6, 6–6.9, 7–7.9, or ≥8 hours/day), obesity, percieved stress (high, medium, or low), medical history of hypertension, diabetes, dyslipidemia, ischemic heart disease, stroke, or cancer, and use of medication for hypertension, diabetes, dyslipidemia, inflammation, constipation, or sleep disorder.

## DISCUSSION

The J-MICC Study’s Saga region cohort enrolled about 12 000 people from the general population in the southern part of Japan.^14^ At the face-to-face 5-year second survey, we found that non-participation was associated with being female, both younger and older age (compared with 60–64 years of age), lower occupational class, current smoking status, lower physical activity level, shorter periods of sleep, obesity, and use of medication for constipation. In contrast, drinking 0.1 to 22.9 g ethanol/day was associated with participation. In addition, individuals who developed cancer during the follow-up period were less likely to participate in the second survey. Notably, the characteristics of non-participants are considered to be potential risk factors for cancer. This finding suggests that the influence of non-participation bias should be considered carefully, because both risk factors and disease might have associations with non-participation. Most large longitudinal studies in Japan have not produced reports that focus on non-participation in second surveys. Assessing differences between participants and non-participants is especially important in longitudinal studies, as missing follow-up data may lead to bias.

The incidence of cancer appeared to influence non-participation in the second survey; however, a past history of cancer at baseline was not found to be associated with participation in a second survey, as previous cohort studies have reported.^4^^,^^10^ There are several possible reasons why patients who develop cancer during follow-up are less likely to participate. For example, cancer symptoms may be too serious or a person’s physical capabilities may be too poor after receiving cancer medication to allow them to participate. Additionally, as the aim of the J-MICC Study is to investigate the prevention of lifestyle-related diseases—mainly cancer—according to genetic traits, people with cancer may think that they are no longer able to participate in the cohort study because they had already reached an endpoint, or that participation in any further follow-up is not needed. It is of serious concern when information on the incidence of cancer is not reported among non-participants in face-to-face second surveys. Therefore, efforts to reduce non-participation should be made, such as providing detailed yet clear information on the requirements of participation and using several follow-up methods. Furthermore, information on the incidence of cancer is better collected from population-based cancer registries or by viewing hospital records, if possible.

The percentage of non-participation in the face-to-face second survey compared to the total baseline population was 25.1% (3029/12 078). In general, participation rates vary by follow-up method, follow-up length, and study population. Attrition from non-participation, other than death or leaving the study area, has been reported to be between 10% and 40%.^2^^–^^7^^,^^9^^,^^11^^,^^12^ Participation rates for face-to-face surveys are reported as being lower than that for mail or telephone surveys, and a shorter time between the first and second assessment is related to a higher participation rate.^4^ With regard to face-to-face surveys, the 5-year EPIC-PANACEA cohort study in Doetinchem (The Netherlands) showed a similar non-participation rate to the current study (22.6%).^4^ Another area of the EPIC-PANACEA cohort study, Cambridge (United Kingdom), had a shorter follow-up length (mean 3.7 years) and showed a higher non-participation rate (39.5%) than the current study. It is therefore possible that the proportion of non-participation varies by study center.

In contrast to the baseline survey,^14^ being female and being in the oldest age group were associated with non-participation in the face-to-face second survey. The characteristics of non-participants in the second survey were inconsistent in previous studies,^3^^–^^5^^,^^7^^,^^10^^,^^12^ and seemed to depend on characteristics of the study, including baseline response rates.

Age is one of the strongest predictors of non-participation. Consistent with previous studies,^2^^–^^5^ we found a higher non-participation rate among individuals <60 years old at the baseline survey. It is possible that such people may be busy with work and housework and may have less time to participate or may have less interest in health-related matters. We also found that the oldest group showed relatively little participation, similar to cohort studies of older populations.^7^^,^^8^ Such people may have developed disease, or may be too frail to participate in face-to-face surveys. The survey method, including methods of announcement and convenience of survey sites, needs to be considered to reduce non-participation. Consistent with other studies,^2^^–^^4^^,^^7^^,^^9^^–^^11^ lower educational and occupational levels were associated with non-participation in the current study.

Similar to previous reports, characteristics associated with a less healthy lifestyle, such as obesity, smoking, lower physical activity, and shorter sleep time, were also associated with non-participation.^2^^–^^5^^,^^7^^,^^9^^,^^10^^,^^12^ Light-to-moderate drinkers tended to participate in our study. It has been reported that light-to-moderate drinking among Japanese drinkers is associated with greater emotional support from friends and co-workers,^16^ and that light-to-moderate alcohol intake is a tool of socialization^17^ and related to good mental health.^18^ Light-to-moderate drinkers in the current study are considered to be more sociable, which may have encouraged them to participate. Although previous studies have reported that heavy alcohol intake was associated with non-participation,^3^^,^^4^^,^^10^ such an association was not evident in males but did show a tendency in females in the current study.

Medical history and use of medication at baseline were not associated with participation, except for medication for constipation. Constipation is a functional gastrointestinal disorder, and is associated with reduced quality of life and psychological disorders.^19^^,^^20^ Therefore, correlations between constipation and psychological factors might have an association with participation.

An interesting aspect of the current study is that self-reported disease development, other than stroke in females, was not associated with the response to the survey method (mail or telephone) among people who did not participate in the face-to-face survey. This suggests that a telephone survey may be a useful way not only to ascertain disease development among young or obese people, who are less likely to answer a mail survey, but also to detect people who have had a stroke during the follow-up period.

This study has several limitations. First, most of the factors related to non-participation are based on baseline data, while some lifestyle-related factors might change over time. Second, incidence of disease was based on a self-report. Also, we had no information on disease development for 410 participants who had not given their clinical history by mail or telephone surveys. Further verification using a disease registry, such as a cancer registry, is needed. Third, this cohort is a group highly motivated to participate in the study, and therefore any generalizations should be made with caution.

In conclusion, non-participation in a second survey was associated with gender, age, socioeconomic characteristics, lifestyle behaviors, and health conditions assessed at the baseline survey, and participation may be influenced by disease development during follow-up. To reduce bias introduced by non-participation, completeness of the follow-up survey should be prioritized, and verification of health status using a disease registry or hospital database is necessary.

## ONLINE ONLY MATERIALS

eTable 1. Baseline characteristics of 11 482 participants in the J-MICC Study Saga region by participation status in the face-to-face follow-up survey, and odds ratios and 95% confidence intervals of non-participation according to these characteristics, stratified by gender.

eTable 2. Baseline characteristics of 2608 people who did not participate in the face-to-face follow-up survey but participated in the health survey, by response status (mail or telephone), and odds ratios and 95% confidence intervals of telephone responses according to these characteristics, stratified by gender.

Abstract in Japanese.
