# Supplementary material for: Factors Associated With Non-participation in a Face-to-Face Second Survey Conducted 5 Years After the Baseline Survey
Source: J Epidemiol. 2015 Feb 5;25(2):117–25. doi: 10.2188/jea.JE20140116 (PMC4310872; doi:10.2188/jea.JE20140116)
Supplement: eTable 1. [file je-25-117-s001.pdf]

eTable 1. Baseline characteristics of 11,482 participants in the J-MICC Study Saga region by participation status in the face-to-face follow-up survey, and odds ratios and 95% confidence intervals of non-participation according to these characteristics, stratified by gender

Baseline characteristics		Male (n=4,755)				Female (n=6,727)			
		Non-participants	Participants	OR[a]	(95% CI)	Non-participants	Participants	OR[a]	(95% CI)
Number		1,259	3,496			1,769	4,958		
Age category	40-44 years	182	333	2.02	(1.56 - 2.62)	232	635	1.40	(1.12 - 1.74)
	45-49 years	173	376	1.76	(1.36 - 2.28)	266	641	1.54	(1.24 - 1.91)
	50-54 years	196	539	1.31	(1.03 - 1.67)	295	806	1.32	(1.08 - 1.62)
	55-59 years	237	752	1.13	(0.90 - 1.41)	334	1,022	1.16	(0.96 - 1.40)
	60-64 years	224	753	1.00	(ref)	300	1,030	1.00	(ref)
	65-69 years	247	743	1.07	(0.85 - 1.33)	342	824	1.34	(1.10 - 1.61)
Education, years (≤12 vs. >12)		704	1,728	1.26	(1.10 - 1.46)	1,122	2,877	1.20	(1.06 - 1.36)
Occupational class	High	466	1,340	1.00	(ref)	275	859	1.00	(ref)
	Medium	310	941	0.942	(0.79 - 1.12)	538	1,471	1.11	(0.93 - 1.32)
	Low	258	575	1.19	(0.98 - 1.45)	201	467	1.36	(1.08 - 1.71)
	None	223	634	1.04	(0.83 - 1.30)	734	2,114	1.05	(0.88 - 1.26)
Drinking status	Never	245	562	1.00	(ref)	1,022	2,767	1.00	(ref)
	Former	49	113	0.84	(0.56 - 1.25)	53	102	1.11	(0.78 - 1.58)
	0.1-22.9 g ethanol/day	400	1,304	0.78	(0.64 - 0.95)	588	1,881	0.86	(0.76 - 0.97)
	23.0-45.9 g ethanolday	280	787	0.86	(0.69 - 1.06)	64	146	1.08	(0.78 - 1.48)
	≥46 g ethanol/day	283	728	0.86	(0.69 - 1.07)	41	57	1.36	(0.87 - 2.12)
Smoking status	Never	257	893	1.00	(ref)	1,455	4,385	1.00	(ref)
	Former	446	1,439	1.07	(0.90 - 1.29)	98	228	1.18	(0.92 - 1.53)
	Current	556	1,164	1.47	(1.23 - 1.77)	216	344	1.65	(1.36 - 2.00)
Quartiles of PAL	Q1 (<1.402)	415	977	1.33	(1.10 - 1.60)	439	936	1.64	(1.38 - 1.94)
	Q2 (1.402-1.449)	278	850	1.06	(0.87 - 1.29)	471	1,287	1.33	(1.13 - 1.56)
	Q3 (1.450-1.505)	246	768	1.01	(0.82 - 1.23)	429	1,329	1.20	(1.02 - 1.41)
	Q4 (≥1.506)	277	854	1.00	(ref)	393	1,365	1.00	(ref)
Sleeping category (hours)	<6	128	289	1.34	(1.05 - 1.71)	288	726	1.11	(0.94 - 1.33)
	≥6 to <7	389	1,085	1.10	(0.93 -1.30)	642	1,888	0.99	(0.87 - 1.14)
	≥7 to <8	433	1,345	1.00	(ref)	602	1,738	1.00	(ref)
	≥8	308	776	1.22	(1.02 - 1.47)	235	605	1.04	(0.87 - 1.26)
BMI category (kg/m^2)	<18.5	40	102	0.93	(0.62 - 1.38)	145	413	1.00	(0.81 - 1.23)
	≥18.5 to <25	803	2,368	1.00	(ref)	1,206	3,742	1.00	(ref)
	≥25	412	1,022	1.15	(0.99 - 1.33)	417	799	1.58	(1.37 - 1.82)
Percieved stress	High	331	796	1.08	(0.89 - 1.31)	605	1,608	1.03	(0.87 - 1.22)
	Medium	567	1,651	0.96	(0.82-1.13)	852	2,421	1.01	(0.87 - 1.19)
	Low	359	1,047	1.00	(ref)	312	926	1.00	(ref)
Medical history[b]	Hypertension	321	831	1.30	(0.99 - 1.70)	316	752	0.81	(0.61 - 1.09)
	Diabetes	144	336	1.10	(0.78 - 1.56)	85	172	1.59	(1.00 - 2.54)
	Dyslipidemia	261	761	0.97	(0.80 - 1.18)	309	887	0.97	(0.81 - 1.18)
	Ischemic heart disease	50	158	0.83	(0.63 - 1.15)	43	105	0.99	(0.68 - 1.45)
	Stroke	37	81	1.25	(0.81 - 1.93)	21	43	1.09	(0.62 - 1.90)
	Cancer	62	184	1.05	(0.77 - 1.44)	102	264	1.05	(0.82 - 1.34)
On medication[c]	Hypertension	252	696	0.85	(0.63 - 1.15)	274	589	1.52	(1.11 - 2.08)
	Diabetes	97	215	1.17	(0.76 - 1.78)	55	118	0.75	(0.43 - 1.33)
	Dyslipidemia	114	312	1.06	(0.79 - 1.41)	181	510	0.89	(0.69 - 1.13)
	Anti-inflammatory drugs	44	116	1.01	(0.69 - 1.49)	106	219	1.24	(0.97 - 1.60)
	Constipation	44	62	1.76	(1.14 - 2.72)	168	361	1.23	(1.00 - 1.50)
	Sleeping disorder	50	118	1.01	(0.71 - 1.45)	116	234	1.21	(0.94 - 1.55)

BMI, body mass index; CI, confidence interval; OR, odds ratio; PAL, physical activity level, which was calculated as total daily energy expenditure divided by b

[a] Adjusted for all items listed in the table

[b] Compared to no history of disease
