# Supplementary material for: Factors Associated With Non-participation in a Face-to-Face Second Survey Conducted 5 Years After the Baseline Survey
Source: J Epidemiol. 2015 Feb 5;25(2):117–25. doi: 10.2188/jea.JE20140116 (PMC4310872; doi:10.2188/jea.JE20140116)
Supplement: eTable 2. [file je-25-117-s002.pdf]

eTable 2. Baseline characteristics of 2,608 people who did not participate in the face-to-face follow-up survey but participated in the health survey, by response status (mail or telephone), and odds ratios and 95% confidence intervals of telephone responses according to these characteristics, stratified by gender

Baseline characteristics		Male (n=1,079)				Female (n=1,529)			
		Telephone survey	Mail survey	Adjusted OR[a]	(95% CI)	Telephone survey	Mail survey	Adjusted OR[a]	(95% CI)
Number		288	791			260	1,269		
Age category	40-44 years	70	83	3.25	(1.88-5.62)	40	148	1.70	(0.98-2.95)
	45-49 years	51	92	1.99	(1.14-3.46)	41	182	1.40	(0.82-2.39)
	50-54 years	51	122	1.56	(0.92-2.63)	44	216	1.19	(0.72-1.97)
	55-59 years	50	161	1.12	(0.67-1.88)	55	240	1.31	(0.82-2.09)
	60-64 years	37	155	1.00	(ref)	40	225	1.00	(ref)
	65-69 years	29	178	0.74	(0.41-1.30)	40	258	0.88	(0.53-1.45)
Education, years (≤12 vs. >12)		172	429	1.54	(1.11-2.13)	159	816	0.95	(0.70-1.28)
Occupational class	High	120	285	1.00	(ref)	44	182	1.00	(ref)
	Medium	72	193	0.91	(0.63-1.33)	81	385	0.84	(0.55-1.30)
	Low	70	150	1.07	(0.71-1.63)	33	147	1.05	(0.61-1.79)
	None	25	163	0.53	(0.29-0.96)	101	539	0.83	(0.54-1.26)
Drinking status	Never	64	133	1.00	(ref)	155	734	1.00	(ref)
	Former	9	30	0.66	(0.26-1.67)	8	37	0.98	(0.43-2.25)
	0.1-22.9 g ethanol/day	87	264	0.63	(0.41-0.97)	82	427	0.87	(0.64-1.19)
	23.0-45.9 g ethanolday	63	183	0.64	(0.40-1.00)	11	41	1.03	(0.49-2.19)
	≥46 g ethanol/day	64	180	0.57	(0.36-0.91)	4	29	0.53	(0.18-1.62)
Smoking status	Never	50	169	1.00	(ref)	210	1,050	1.00	(ref)
	Former	97	296	1.16	(0.76-1.77)	8	77	0.49	(0.23-1.06)
	Current	141	326	1.19	(0.78-1.80)	42	142	1.51	(1.02-2.26)
Quartiles of PAL	Q1 (<1.402)	81	271	1.01	(0.67-1.52)	62	317	1.34	(0.87-2.07)
	Q2 (1.402-1.449)	63	187	0.94	(0.61-1.44)	73	332	1.33	(0.88-1.99)
	Q3 (1.450-1.505)	63	138	1.35	(0.87-2.10)	64	313	1.17	(0.78-1.76)
	Q4 (≥1.506)	72	177	1.00	(ref)	55	286	1.00	(ref)
Sleeping category (hours)	<6	31	70	1.15	(0.68-1.95)	40	202	0.94	(0.60-1.46)
	≥6 to <7	108	230	1.31	(0.92-1.87)	103	450	1.12	(0.80-1.57)
	≥7 to <8	94	273	1.00	(ref)	85	445	1.00	(ref)
	≥8	54	218	0.90	(0.59-1.37)	31	171	1.02	(0.63-1.64)
BMI category (kg/m^2)	<18.5	8	23	1.43	(0.57-3.58)	8	107	0.36	(0.17-0.75)
	≥18.5 to <25	166	535	1.00	(ref)	178	874	1.00	(ref)
	≥25	113	231	1.57	(1.15-2.14)	74	287	1.36	(0.98-1.88)
Percieved stress	High	82	202	1.09	(0.71-1.69)	97	411	1.05	(0.69-1.59)
	Medium	147	341	1.50	(10.3-2.18)	115	625	0.87	(0.59-1.29)
	Low	59	246	1.00	(ref)	48	233	1.00	(ref)
Medical history[b]	Hypertension	56	227	0.61	(0.33-1.13)	48	228	0.77	(0.38-1.60)
	Diabetes	29	85	0.96	(0.39-2.36)	18	53	3.10	(1.23-7.82)
	Dyslipidemia	59	167	1.17	(0.76-1.79)	52	228	1.15	(0.70-1.77)
	Ischemic heart disease	9	32	1.3	(0.56-3.06)	4	30	0.69	(0.23-2.08)
	Stroke	6	26	1.22	(0.45-3.31)	4	14	1.56	(0.47-5.19)
	Cancer	9	42	0.79	(0.36-1.76)	14	72	0.99	(0.53-1.86)
On medication[c]	Hypertension	46	178	1.27	(0.65-2.48)	43	196	1.35	(0.63-2.91)
	Diabetes	21	57	1.75	(0.61-5.03)	9	38	0.42	(0.13-1.36)
	Dyslipidemia	19	78	0.72	(0.36-1.41)	31	131	1.10	(0.61-2.00)
	Anti-inflammatory drugs	14	24	1.97	(0.91-4.27)	15	70	1.03	(0.56-1.91)
	Constipation	6	30	0.78	(0.27-2.30)	23	119	1.01	(0.62-1.65)
	Sleeping disorder	11	30	1.48	(0.67-3.24)	11	85	0.59	(0.29-1.20)

BMI, body mass index; CI, confidence interval; OR, odds ratio; PAL, physical activity level, which was calculated as total daily energy expenditure divided by

[a] Adjusted for all items listed in the table

[b] Compared to no history of disease
