# Supplementary material for: Factors Associated With Non-participation in a Face-to-Face Second Survey Conducted 5 Years After the Baseline Survey
Source: J Epidemiol. 2015 Feb 5;25(2):117–25. doi: 10.2188/jea.JE20140116 (PMC4310872; doi:10.2188/jea.JE20140116)
Supplement: Abstract in Japanese. [file je-25-117-s003.pdf]

ベースライン調査から5年後の対面による第二次調査への非参加に関連する要因

原めぐみ[1]、島ノ江千里[1]、大塚康子[1]、西田裕一郎[1]、南里妃名子[2]、堀田美加子[1]、安方淳[3]、三好伸幸[4]、山田陽介[5]、檜垣靖樹[3]、田中恵太郎[1]

[1]佐賀大学医学部社会医学講座予防医学

[2]昭和大学医学部衛生学公衆衛生学講座公衆衛生学

[3]福岡大学スポーツ科学部

[4]精華女子短期大学幼児保育学科

[5]国立健康・栄養研究所基礎栄養研究部エネルギー代謝研究室

背景：第二次調査への参加状況はベースライン時点の特性と関連があると報告されているが、日本人についての報告はない。また、追跡期間中の疾患罹患も第二次調査への参加状況に影響すると考えられるが、報告は少ない。本研究では、住民ベースのコーホート研究の第二次調査への非参加に関連するベースライン時点の要因を明らかにするとともに、追跡期間中の疾病罹患の影響について評価した。

方法：2005 年から 2007 年にかけて実施された日本多施設共同コーホート研究佐賀地区に参加した 12,078 人の参加者から、追跡期間中の転出（423 人）、死亡（163 人）、同意撤回（9 人）を除いた 11,483 人を対象に、2010 年から 2012 年にかけて対面式の第二次調査を実施した。非参加者については、郵送法または電話で 5 年間の疾患罹患を調査した。非参加者のベースライン時点の特性、および自己申告の追跡期間中の疾患罹患状況について参加者と比較した。

結果：11,483 人のうち 8,454 人（73.6%）が第二次調査に参加し、非参加者 3,029 人のうち 2,608 人が郵送法または電話で罹患状況について回答した。ベースライン調査時点の性（女性）、年齢（若い群と最も高齢群）、短い教育歴、低い職業クラス、喫煙、低い身体活動レベル、短い睡眠時間、肥満、便秘が第二次調査への非参加と、適量飲酒（1日エタノール摂取量 23 g 未満）が参加と関連していた。非参加者は参加者に比べて、追跡期間中のがん罹患割合が高く、高血圧の発症割合が低かった。

結論：ベースライン調査時点の特性と追跡期間中の罹患状況の両方が、対面式の第二次調査への参加状況と有意に関連していた。

キーワード：非参加、第二次調査、コーホート、社会経済因子、生活習慣関連因子
